# Delayed and Transient Increase of Adult Hippocampal Neurogenesis by Physical Exercise in DBA/2 Mice

**DOI:** 10.1371/journal.pone.0083797

**Published:** 2013-12-20

**Authors:** Rupert W. Overall, Tara L. Walker, Odette Leiter, Sina Lenke, Susann Ruhwald, Gerd Kempermann

**Affiliations:** 1 CRTD - Center for Regenerative Therapies Dresden, Genomics of Regeneration, Technische Universität Dresden, Dresden, Germany; 2 German Center for Neurodegenerative Diseases (DZNE), Dresden, Dresden, Germany; Seattle Children's Research Institute, United States of America

## Abstract

This study builds on the findings that physical activity, such as wheel running in mice, enhances cell proliferation and neurogenesis in the adult hippocampus of the common mouse strain C57BL/6, and that the baseline level of neurogenesis varies by strain, being considerably lower in DBA/2. Because C57BL/6 and DBA/2 are important as the parental strains of the BXD recombinant inbred cross which allows the detection of genetic loci regulating phenotypes such as adult neurogenesis, we performed the current study to investigate the gene x environment interactions regulating neurogenesis. At equal distances and times run DBA/2J mice lacked the acute increase in precursor cell proliferation known from C57BL/6. In DBA/2J proliferation even negatively correlated with the distance run. This was neither due to a stress response (to running itself or single housing) nor differences in estrous cycle. DBA/2 animals exhibited a delayed and weaker pro-neurogenic response with a significant increase in numbers of proliferating cells first detectable after more than a week of wheel running. The proliferative response to running was transient in both strains, the effect being undetectable by 6 weeks. There was also a small transient increase in the production of new neurons in DBA/2J, although these extra cells did not survive. These findings indicate that the comparison between C57BL/6 and DBA/2, and by extension the BXD genetic reference population derived from these strains, should provide a powerful tool for uncovering the complex network of modifier genes affecting the activity-dependent regulation of adult hippocampal neurogenesis. More generally, our findings also describe how the external physical environment interacts with the internal genetic environment to produce different responses to the same behavioral stimuli.

## Introduction

Adult neurogenesis, the generation of new neurons throughout life, occurs in the dentate gyrus of the hippocampus in all mammalian species investigated so far [1], including, of course, mice. Adult neurogenesis consists of several partially overlapping and independently regulated steps [2,3]. Among these, the initial proliferation of neural precursor cells is dynamically regulated by extrinsic and behavioral stimuli—most strikingly physical exercise [4]. Baseline levels of precursor proliferation also vary enormously among different strains of mice [5-8]. The interaction between genetic background and physical activity in modulating levels of adult neurogenesis, however, has been less well studied—although strain-specific differences in the effect of wheel running on maturation of new neurons have been reported [9]. Relatedly, we have previously reported that environmental enrichment has a robust pro-neurogenic effect on 129/SvJ mice, which have an extremely low baseline level of neurogenesis and that in this case environmental enrichment also increased precursor cell proliferation, an effect that is essentially absent in C57BL/6 at this stage [10]. These results suggest that latent genetic variation could yield clues to the molecular mechanisms coupling physical activity and neural precursor proliferation. 

C57BL/6 and DBA/2, the two mouse strains used in the current study, have been shown to differ strikingly in both hippocampal precursor cell proliferation [6] and the net production of new neurons [6,7,9]. These two strains also differ in many other traits and are the parental strains for the BXD panel of recombinant inbred lines. The BXD panel has been extensively studied including with respect to adult neurogenesis [7] and provides a powerful tool for the dissection of the genetics of complex traits [11]. Large variation across the BXD lines has also been demonstrated for a number of relevant phenotypes such as volume of hippocampal subregions [12], water maze performance [6], adult hippocampal neurogenesis [7] as well as in hippocampal transcript expression [13].

In this study, we investigated proliferation responses to wheel running in DBA/2 mice with comparison to C57BL/6. We focused on key time points that are known to be associated with a strong response in C57BL/6 animals. We hypothesized that, in addition to the previously reported difference in baseline adult neurogenesis, DBA/2 mice might also differ in their response to physical activity, and would thus present a useful model for the study of the genetics of behaviorally induced brain plasticity. 

## Methods

### Ethics Statement

All experiments were conducted in accordance with the applicable European and National regulations (Tierschutzgesetz) and approved by the responsible authority (Regierungspräsidium Dresden; Permit Number: 24-9168.11-1/2009-42).

### Animals and housing

Female mice of the strains DBA/2JRj and C57BL/6JRj were purchased from Janvier and subsequently housed at the animal facility of the Medizinisch-Theoretisches Zentrum at Technische Universität Dresden, Germany. Standard mouse chow and water were provided *ad libitum*. Animals were housed two per cage (except for the single-housed 4 day groups) in standard polycarbonate cages (Type III, Tecniplast, Germany) with or without a running wheel (150 mm diameter, TSE Systems, Germany) and a 12 h light/dark cycle. Wheels were fitted with electronic counters and were monitored for the duration of the experiment. For analysis, total wheel revolutions during the hours of darkness were counted and converted into distance run. To label proliferating cells, BrdU (50 mg/kg body weight; Sigma) or IdU (57.5 mg/kg body weight) was injected intraperitoneally at 9 pm during the animals’ active phase and the mice were killed 12 h later. For measures of precursor survival, a single intraperitoneal injection of CldU (42.5 mg/kg body weight; Sigma) was given in the afternoon as the animals were introduced into the experimental housing. For the cumulative labeling, 10 injections of IdU (57.5 mg/kg body weight; Sigma) were administered in the afternoon of the first 10 days of the experiment. All animals were 10 weeks old on day 0 of the respective experimental period.

### Determination of estrus cycle

For the 4-day single-housed groups, the estrus cycle at the time of the BrdU injection was determined by vaginal smear cytology [14]. Briefly, the animal’s vagina was flushed with a drop of saline which was smeared onto a glass slide. Cell type and number were observed under a microscope and classified into one of four stages. Proestrus was characterized by a predominance of nucleated epithelial cells, estrus by clusters of anucleated cornified cells, and diestrus by the prevalence of leucocytes. Metestrus was distinguished by the presence of all three types of cell.

### Corticosterone assay

Blood used for assessment of corticosterone levels was collected from the atrial incision during perfusion. Collection took place between 9–10 AM, approximately 2–3 hours after onset of the light cycle. Blood was processed using the Corticosterone EIA Kit (Enzo Life Sciences, Germany) following the manufacturer’s protocol. Assays were read using a Nanoquant Infinite M200 ELISA plate reader (Tecan, Switzerland). Data were interpolated from the standard curve using a 4-parameter curve fitting script written in R.

### Histology

Histology for BrdU, CldU and IdU detection was carried out using standard protocols [15]. Briefly; animals were transcardially perfused with 0.9 % NaCl and the brain removed. For this study, one randomly selected hemisphere was post-fixed overnight in a 4 % solution of paraformaldehyde and stored in cryoprotectant solution. 40 µm coronal sections were cut on a freezing microtome and every sixth section was first washed twice with 0.9 % NaCl then treated with 2 N HCl for 30 min at 37 °C. Multiple washes in phosphate-buffered saline were performed between all further steps. After blocking with 10 % donkey serum containing 0.2 % Triton X-100, sections were incubated overnight with primary antibodies (for CldU or BrdU labeling: rat anti-BrdU, AbD Serotec, Germany; for IdU labeling: mouse anti-BrdU, BD Biosciences, Germany; for NeuN: rabbit anti-FOX3/NeuN, Abcam, UK) in blocking solution containing 3 % donkey serum and 0.2 % Triton X-100. BrdU samples were detected with a biotinylated anti-rat secondary (Vector Laboratories, USA) together with the horseradish peroxidase-coupled ABC Elite system (Vector Laboratories, USA) and visualized with 3,3′-diaminobenzidine before counting under a light microscope. CldU, IdU, and NeuN triple-labeled samples were detected with fluorescent secondary antibodies (donkey anti-rat Alexa Fluor 488, donkey anti-mouse Cy3 and donkey anti-rabbit Alexa Fluor 647; Jackson ImmunoResearch, UK), the nuclei counterstained with 4′,6-diamidino-2-phenylindole, and then directly visualized for counting using our standard methodology as described and discussed elsewhere [16,17]. This method is a simplified version of the optical fractionator principle. Briefly, all labeled (BrdU^+^, IdU^+^ or CldU^+^) cells in the subgranular zone and the granule cell layer were counted except for cells in the uppermost focal plane (at 40x magnification) which were disregarded to avoid oversampling at the cutting surfaces. CldU^+^NeuN^+^ double-labeled cells were analyzed using an ApoTome fluorescence microscope (Zeiss, Germany) with Optical Sectioning mode (Structured Illumination Microscopy). Each CldU^+^ cell was individually imaged at 40x magnification as an optical section and 100 of these cells for each animal were randomly selected and examined for NeuN immunoreactivity. All counts were carried out with the experimenter blind to the experimental group.

### Statistical analysis

All analyses were carried out using the statistical software R/Bioconductor. All *t*-tests were unpaired, two-tailed and assumed equal variances. The effects of estrus cycle were tested using a one-way ANOVA model. As both ANOVA tests were not significant, post-hoc testing was not justified and therefore not performed. Corticosterone data were log-transformed for statistical analysis. Normalized difference from control (NDC) values for the summary graph were calculated as (RUN - STD) / STD which yields a symmetric representation of the amount of change from 0 (where RUN = STD). Error bars in the summary graph were calculated by propagation of the standard error from STD and RUN groups. 

## Results

### DBA/2 mice do not show an increase in hippocampal neural precursor proliferation after short-term running

**Figure 1:**
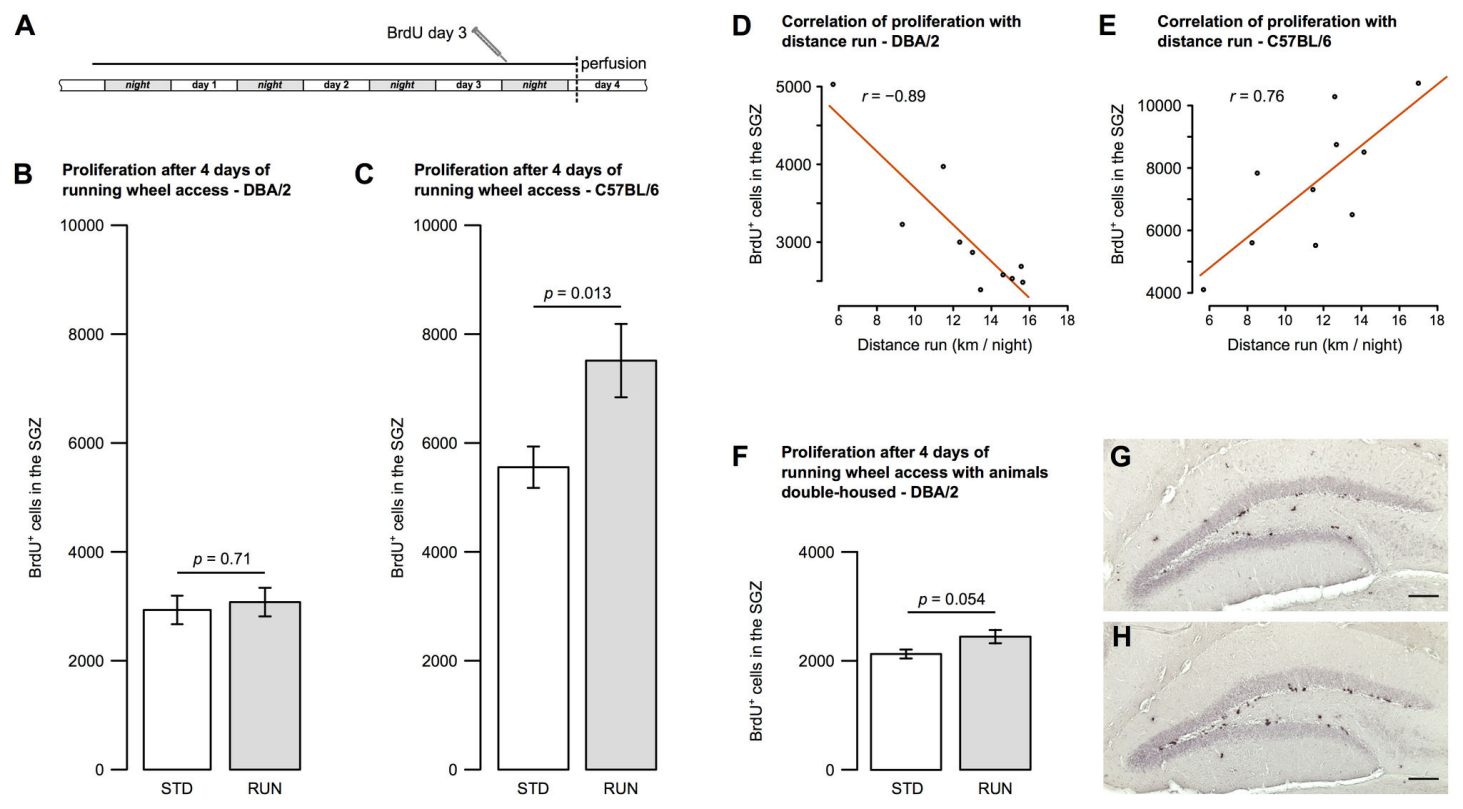
Precursor cell proliferation is affected differently in C57BL/6 and DBA/2 by short-term wheel running. Proliferating cells in the subgranular zone were measured by incorporation of BrdU 12 h before experiment end (**A**). Data for single-housed mice showed no change in proliferating cell numbers after 4 days running in DBA/2 (**B**) but an increase in C57BL/6 (**C**). Proliferation correlated negatively with running wheel use in the DBA/2 animals (**D**), but positively for the C57BL/6 mice (**E**). Housing the DBA/2 mice in pairs also did not lead to a significant increase in proliferation after wheel running (**F**). Representative BrdU-stained sections are shown from C57BL/6 standard-housed (**G**) and running (**H**) animals. Bar graphs show mean ± SEM. All *p*-values are from two-tailed Student’s *t*-tests. Scale bars in G and H are 100 µm.

**Figure 2:**
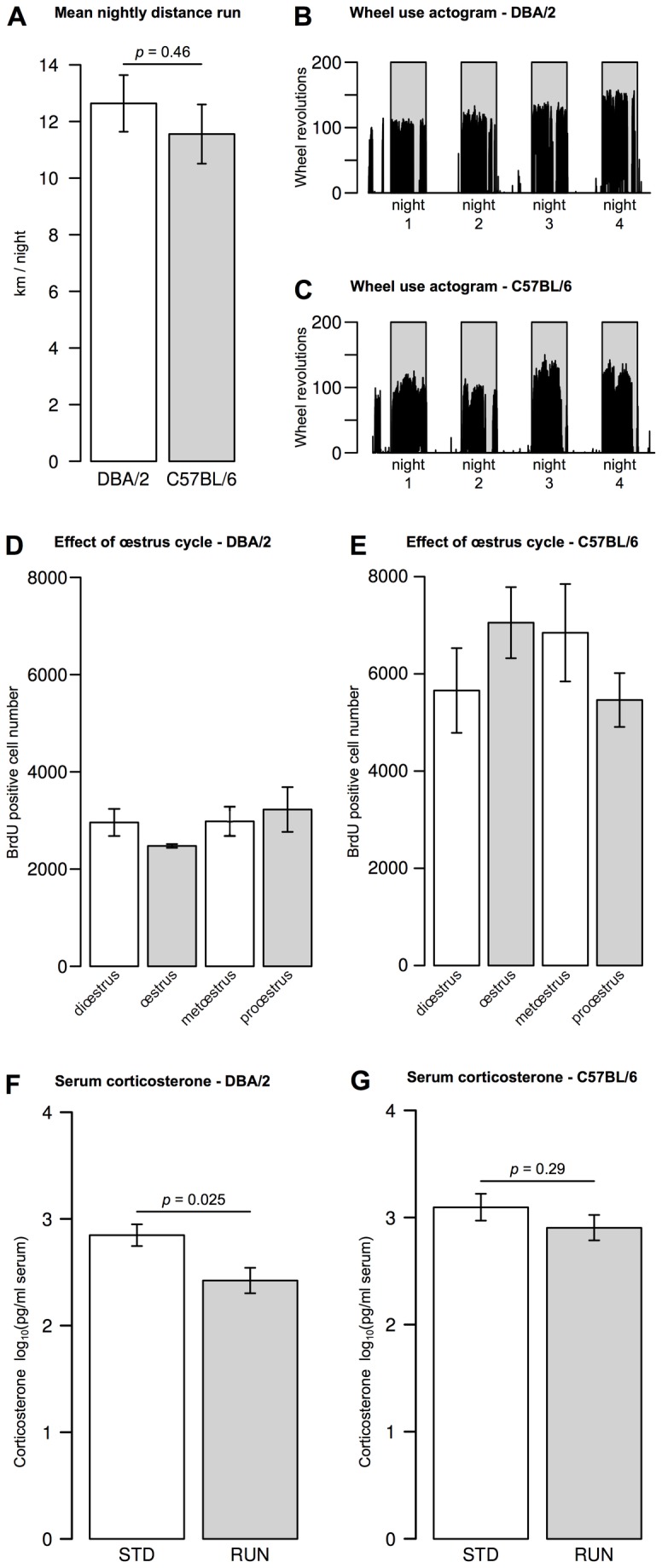
Potential confounding factors. DBA/2 mice did not run significantly different distances (**A**) or exhibit different running patterns. Typical actograms are shown for DBA/2 (**B**) and C57BL/6 (**C**) demonstrating that both strains, after a burst of activity as they are introduced to the new cage, run almost continuously throughout the hours of darkness (shaded regions). No significant effect of estrus cycle was observed in DBA/2 (**D**) or C57BL/6 (**E**) following one-way ANOVA. Serum corticosterone levels were reduced by running in DBA/2 (**F**) but not significantly in C57BL/6 (**G**). Bar graphs show mean ± SEM. All *p*-values are from two-tailed Student’s *t*-tests.

When compared with C57BL/6 controls, the DBA/2 mice in standard housing exhibited a consistently lower baseline number of proliferating hippocampal precursor cells—a difference which has been noted previously [6]. An increase in precursor cell proliferation in C57BL/6 mice housed with running wheels has been consistently reported [4,9,17-21]. Surprisingly, however, we found that the effect of physical activity on precursor proliferation was absent in DBA/2 after 4 days of running wheel activity (STD 2933 ± 262, RUN 3077 ± 262, all results reported as number of positive cells per both hippocampi ± SEM, *t*(22) = 0.38, *p* = 0.71; Figure **1A, B** and Table **1**). This was in contrast to our confirmation of an exercise-induced increase in proliferation in C57BL/6 (STD 5555 ± 380, RUN 7513 ± 674, *t*(22) = 2.71, *p* = 0.013; Figure **1C** and Table **1**). In addition, using revolution counters on the running wheels, we observed that running in C57BL/6 appears to have a quantitative effect, with proliferating cell numbers increasing in proportion to running wheel usage (Pearson’s r = 0.76, *p* = 0.01). Interestingly, however, this was not the case for DBA/2 where the distance run even exhibited a negative correlation with BrdU-labeled cell counts (Pearson’s r = -0.89, *p* = 0.0005; Figure **1D, E**). The actograms also confirmed that mice of the two strains use the wheels in a similar pattern with an indistinguishable distance run over the 4 day period (DBA/2: 12.62 ± 3.14 km / night, C57BL/6: 11.54 ± 3.28 km / night, *t*(18) = 0.75, *p* = 0.46; Figure **2 A-C**). 

**Table 1:** Summary of histological data for all groups.

**Group**	**Genotype**	**Duration**	**BrdU^*+*^**	**IdU^*+*^**	**CldU^*+*^**	**Labeled NeuN^*+*^**	**N**
STD	DBA/2	4 day	2933 ± 262				14
RUN	DBA/2	4 day	3077 ± 262				10
STD*	*DBA/2*	*4 day*	*2122 ± 87*				*8*
RUN*	*DBA/2*	*4 day*	*2445 ± 127*				*8*
STD	C57BL/6	4 day	5555 ± 380				14
RUN	C57BL/6	4 day	7513 ± 674				10
STD	DBA/2	14 day		3139 ± 221	1921 ± 332	1678 ± 308	10
RUN	DBA/2	14 day		4022 ± 333	2329 ± 295	1968 ± 251	10
STD	DBA/2	21 day		2072 ± 142	1378 ± 199	1026 ± 171	10
RUN	DBA/2	21 day		2804 ± 213	1924 ± 200	1675 ± 192	10
STD	DBA/2	28 day		2287 ± 112	2170 ± 277	1578 ± 235	10
RUN	DBA/2	28 day		2832 ± 139	2339 ± 220	1544 ± 117	9
STD	C57BL/6	28 day		3528 ± 146	2813 ± 193	2128 ± 154	10
RUN	C57BL/6	28 day		4116 ± 176	3587 ± 183	3020 ± 209	10
STD	DBA/2	42 day		2152 ± 247	2440 ± 396	1833 ± 367	10
RUN	DBA/2	42 day		4086 ± 453	2501 ± 118	1969 ± 90	10
STD	C57BL/6	42 day		2991 ± 190	1892 ± 296	1761 ± 280	9
RUN	C57BL/6	42 day		3494 ± 360	3679 ± 375	3574 ± 371	10
STD**	*DBA/2*	*42 day*		*2174 ± 448*		*2062 ± 448*	*10*
RUN**	*DBA/2*	*42 day*		*5864 ± 383*		*5820 ± 385*	*10*

Histological counts for all groups including raw survival data and group sizes (column “N”). The column “labeled NeuN+” contains counts of NeuN+ cells which were also double-positive for CldU or IdU (depending on the group). Values are given as mean ± standard error of the mean. *4-day double housing group. **Cumulative labeling protocol.

Because all animals used were females, and there is some evidence that estrogen levels can influence adult neurogenesis [22], we noted the estrous cycle stage of each animal at the start of the 4 day experimental period. Using a one-way ANOVA model, there was no significant effect of estrus cycle stage on BrdU-labeled cell counts in either strain (DBA/2: *F*(3, 20) = 0.47, *p* = 0.71; C57BL/6: *F*(3, 20) = 1.04, *p* = 0.4; Figure **2D, E**). To determine whether wheel running might be perceived as a stressor by DBA/2 mice, we also measured serum corticosterone levels. The DBA/2 animals had generally lower serum corticosterone than C57BL/6, and showed a further decrease in response to wheel running (STD 704 ± 1.3, RUN 264 ± 1.3 pg / ml serum, *t*(8) = 2.76, *p* = 0.025; Figure **2F**). The slight decrease after running in the C57BL/6 mice, on the other hand, was not significant (STD 1249 ± 1.3, RUN 804 ± 1.3 pg / ml serum, *t*(8) = 1.13, *p* = 0.29; Figure **2G**).

### The effect of single housing is not sufficient to explain the lack of proliferative response in DBA/2 mice

It has been suggested that animals housed in isolation experience increased stress levels which can negatively affect hippocampal precursor proliferation [23,24]. Although all other animals in this study were housed two per cage, the initial 4-day experiment used single-housed mice (for both RUN and STD groups) in order to obtain accurate wheel use information. To test whether the lack of proliferative response in running DBA/2 animals might have been due to the effect of single housing alone, we repeated the 4 day experiment with DBA/2 mice housed in pairs. There was a small increase in labeled cells which was, however, also not statistically significant (STD 2122 ± 87, RUN 2445 ± 127, *t*(14) = 2.1, *p* = 0.054; Figure **1F** and Table **1**). This modest effect size and marginal *p*-value mean that the observed difference in exercise-induced proliferation between DBA/2 and C57BL/6 mice cannot be attributed to the effect of single housing.

### The impairment in proliferation seen in DBA/2 is transient

**Figure 3:**
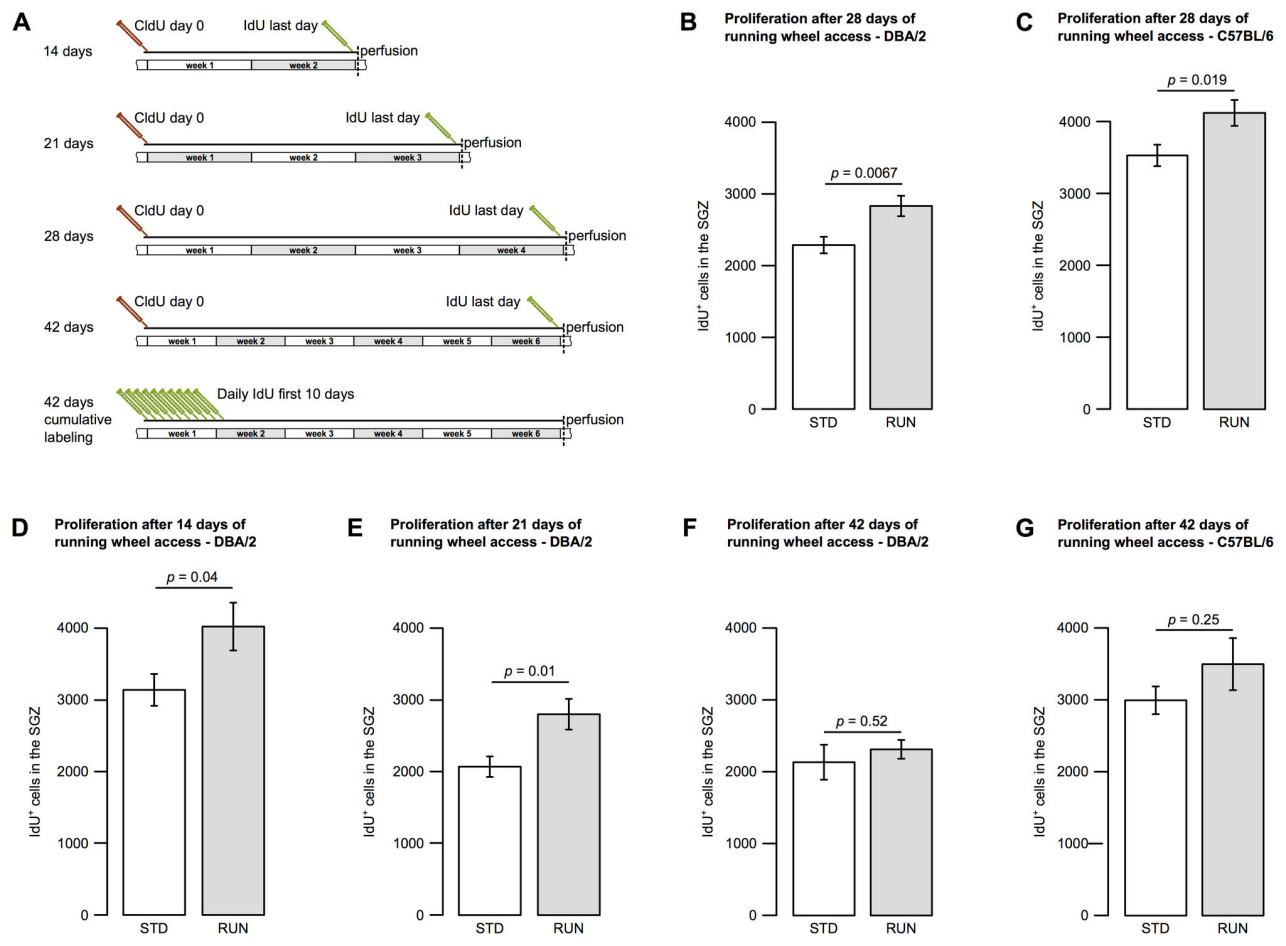
Wheel running induces increased proliferation in DBA/2 animals after two weeks activity. A scheme showing the labeling procedure with IdU (for acute proliferation; green symbol) and CldU (for survival; red symbol) used in this study (**A**). Cells in the subgranular zone proliferating 12 h before experiment end were measured by incorporation of IdU. Animals were housed under standard conditions (STD) or with a running wheel (RUN) for 4 weeks (**B**, **C**), 2 weeks (**D**) or 3 weeks (**E**). After 4 weeks, both DBA/2 (**B**), and C57BL/6 (**C**) mice showed a significant increase in precursor proliferation. DBA/2 animals exhibited a significant pro-proliferative response first after 2 weeks (**D**) and also 3 weeks (**E**) of wheel running. After 6 weeks of continued nightly wheel running, neither DBA/2 (**F**) nor C57BL/6 (**G**) showed a significant increase in proliferating cells in the subgranular zone. Bar graphs show mean ± SEM. All *p*-values are from two-tailed Student’s *t*-tests.

To ascertain whether DBA/2 mice simply require a longer period of exercise for an effect on precursor proliferation, the experiment was also carried out with 28 days of exposure to the running wheel. Taking advantage of the fact that the alternative thymidine analogues IdU and CldU are histochemically distinguishable, we were able to measure proliferation and cell survival in the same animals. To do this, we labeled cells which were dividing at the start of the experiment with a single injection of CldU and assessed the survival of these at experiment end. We also administered IdU on the last day, 12 h before perfusion to obtain a measure of proliferation at that time point (Figure **3A**). 

After 28 days, there was a significant effect of wheel running on precursor cell proliferation, as assessed by the number of IdU-positive cells, in DBA/2 mice (STD 2287 ± 112, RUN 2832 ± 139, *t*(17) = 3.09, *p* = 0.0067) similar to that observed in C57BL/6 animals (STD 3528 ± 146, RUN 4116 ± 176, *t*(18) = 2.57, *p* = 0.019). This finding indicates that the initial lack of the response in DBA/2 was transient (Figure **3B, C** and Table **1**). 

In order to narrow down the time point associated with initiation of running-induced proliferation in DBA/2 mice, animals were also studied after 2 and 3 weeks of exercise. We found that a significant increase in proliferation occurred already after 2 weeks of wheel running (STD 3139 ± 221, RUN 4022 ± 333, *t*(18) = 2.21, *p* = 0.04; Figure **3D** and Table **1**) and remained significant at the 3 week time point (STD 2072 ± 142, RUN 2804 ± 213, *t*(18) = 2.86, *p* = 0.01; Figure **3E** and Table **1**).

These data show that the significant increase in hippocampal precursor proliferation in DBA/2 mice is delayed by about one week compared to animals of the strain C57BL/6, in which most studies have been done to date.

### Sustained wheel running lessens the proliferative response

We also measured precursor proliferation in animals with 6 weeks of continuous access to a running wheel and found that this was not significantly different from the standard-housed mice (DBA/2: STD 2129 ± 240, RUN 2308 ± 128, *t*(18) = 0.66, *p* = 0.52; Figure **3F** and Table **1**). This was also the case for the C57BL/6 mice (C57BL/6: STD 2991 ± 190, RUN 3494 ± 360, *t*(17) = 1.2, *p* = 0.25; Figure **3G** and Table **1**). 

This finding suggests that the pro-proliferative effect of running wheel activity is transient in both strains with, however, different dynamics.

### Physical activity-induced production of new neurons is not generally impaired in DBA/2 mice

New-born neurons in the adult murine hippocampus are thought to require a survival signal from the surrounding niche without which they undergo apoptosis [25,26]. The survival-promoting signal can also affect precursor cells and induce cell cycle exit, and is associated with the onset of maturation into a functional granule cell neuron [27-29]. The genes involved in mediating the survival signaling pathway are known to differ from those controlling proliferation as the two phenotypes are associated with the expression of distinct sets of genes [7]. In C57BL/6 mice, physical activity also results in increased neurogenesis [4]. We therefore investigated whether DBA/2 mice were able to generate new neurons in response to the physical activity stimulus, despite their delayed induction of precursor proliferation. Animals were histologically phenotyped for cells which stained double-positive for both CldU (administered at the beginning of the experiment, see Figure **3A**) and the neuronal marker NeuN (Rbfox3) as a measure of new-born neurons. 

**Figure 4:**
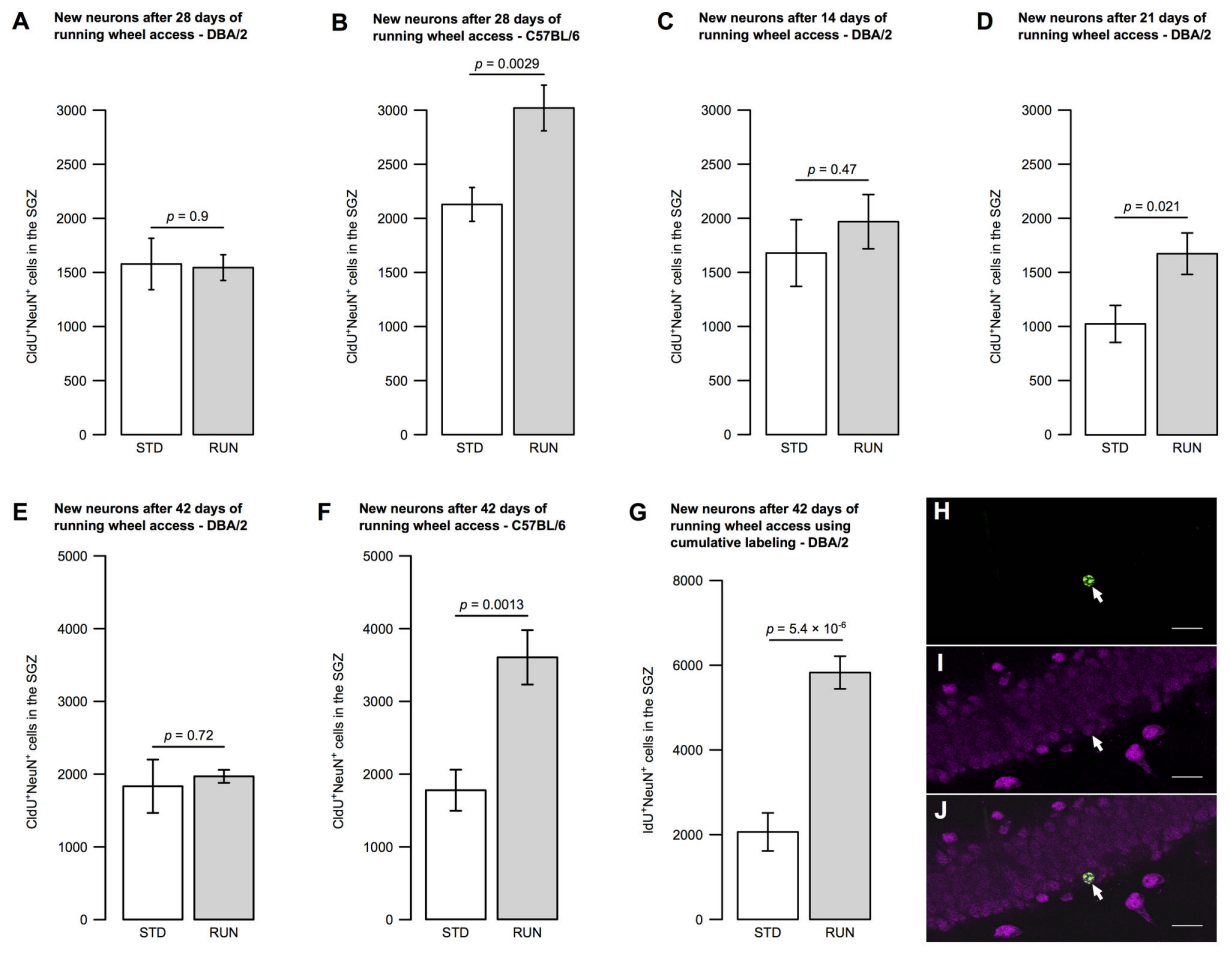
The increase in new-born neuron number by wheel running is delayed in DBA/2 mice. Animals were housed in STD or RUN cages for 4 weeks (**A**, **B**), 2 weeks (**C**), 3 weeks (**D**) or 6 weeks (**E**–**G**). Running mice of the strain C57BL/6 exhibited an increase in new neurons at both time points measured, 4 weeks (**B**) and 6 weeks (**F**). The number of new neurons in DBA/2 mice was only transiently increased after 3 weeks of running and not at 2 weeks (**C**), 4 weeks (**A**) or 6 weeks (**E**) following a single labeling injection. DBA/2 animals labeled with a series of injections over 10 days showed an increased number of new-born neurons after 6 weeks of wheel running (**G**). Representative fluorescence microscopy image of a new-born neuron stained using antibodies against CldU (**H**; green) and NeuN (**I**; magenta) with the arrow indicating a double-positive cell (**J**; merged image). Bar graphs show mean ± SEM. All *p*-values are from two-tailed Student’s *t*-tests. Scale bars in **H–J** are 20 µm.

After 4 weeks of access to a running wheel, DBA/2 mice did not show a significant change in the number of CldU^+^NeuN^+^ new-born neurons in the dentate gyrus (STD 1578 ± 235, RUN 1544 ± 117, *t*(17) = -0.12, *p* = 0.9; Figure **4A** and Table **1**) despite a robust increase in C57BL/6 animals (STD 2128 ± 154, RUN 3020 ± 209, *t*(18) = 3.44, *p* = 0.0029; Figure **4B** and Table **1**). This result would be consistent with the lack of running-induced proliferation seen in DBA/2 animals exposed to fewer than 2 weeks of running activity or, alternatively, due to the lack of a survival response in the new-born immature neurons in DBA/2. To help differentiate between these two scenarios, we also phenotyped surviving CldU-labeled cells in the 2- and 3-week cohorts. We found no significant difference in CldU^+^NeuN^+^ cell counts at 2 weeks (STD 1678 ± 308, RUN 1968 ± 251, *t*(18) = 0.73, *p* = 0.47; Figure **4C** and Table **1**) but, surprisingly, considering the 4-week results, there was an increase in new neurons after 3 weeks of running (STD 1026 ± 171, RUN 1675 ± 192, *t*(18) = 2.53, *p* = 0.021; Figure **4D** and Table **1**).

One might have expected to observe an increase in new neuron production after 6 weeks of running activity, when the majority of the proliferating precursors seen after 2 weeks have had time to differentiate and become mature neurons. We initially carried out this experiment using a single dose of CldU to mark the cells proliferating at day 0 and counted CldU^+^NeuN^+^ new-born neurons after 6 weeks. This labeling approach yielded no significant change in new neuron number in DBA/2 mice (STD 1833 ± 367, RUN 1969 ± 90, *t*(18) = 0.36, *p* = 0.72; Figure **4E** and Table **1**) despite identifying an increase in C57BL/6 animals (STD 1761 ± 280, RUN 3574 ± 371, *t*(17) = 3.83, *p* = 0.0013; Figure **4F** and Table **1**).

A previous study, however, has described just such an increase in new neuron generation in response to 6 weeks of wheel running in a number of mouse strains including DBA/2 [9]. We performed an additional experiment using a cumulative labeling strategy, similar to that used by Clark et al., in which IdU was administered daily over the first 10 days. The results confirmed a substantial increase in new neuron number after wheel running in DBA/2 mice (STD 2062 ± 448, RUN 5820 ± 385, *t*(18) = 6.36, *p* = 5.4 × 10^-6^; Figure **4G** and Table **1**, see also Figure **4H, I, J** for the colocalization).

## Discussion

Although the increase in neural precursor proliferation in response to wheel running in mice has been well established [4,9,17-21], the bulk of such work in mice has been done in one common laboratory strain, C57BL/6. In the present study, we have shown that mice of the strain DBA/2 also exhibit an increase in precursor proliferation after wheel running but that this response is less pronounced and delayed in comparison to C57BL/6. While the level of precursor proliferation increased in C57BL/6 mice in proportion to the distance run, for DBA/2 the inverse was true. This suggests that the delayed effect of physical activity on proliferation in DBA/2 is not due simply to the absence of a response pathway but is, at least in part, the result of a more complex dominant anti-proliferative response in DBA/2 mice to the running induced stimulus. Further work would be required to identify factors which might be transiently regulated in DBA/2 in response to the onset of exercise. 

There have been reports that single-housing might have an adverse effect on adult neurogenesis in the hippocampus [23,24], which has been attributed to social isolation stress. Our results, however indicate that, although numbers of proliferating cells were slightly elevated in DBA/2 mice housed in pairs, this effect was still not significant nor comparable to the effect size seen in C57BL/6 animals. We also noted that DBA/2 mice had slightly lower serum corticosterone than C57BL/6 in concordance with published data [30]. We could also confirm that running reduced corticosterone levels in both strains as has been previously reported [31]—although this was only significant in DBA/2. Corticosterone has been shown to have a negative influence on hippocampal neurogenesis in rodents [23,32]. Thus the absence of a running-induced increase in proliferating precursor cell number in DBA/2, the strain in which serum corticosterone levels were lower and more strongly reduced by wheel running, cannot be explained by differences in corticosterone and is unlikely a stress response. 

The increase in proliferation after physical activity is a transient effect as it was not maintained after 6 weeks of wheel running—which was true for both DBA/2 and C57BL/6. This finding is consistent with our previous report [33] which found no increase in proliferation in male C57BL/6 mice after 32 days of continuous wheel running. Another study, however, showed no increase after only 19 days [34], which our results do not corroborate. The possibility that this decrease itself might be also a transient phenomenon is suggested by further data from the Kronenberg study showing that mice with running-wheel access for 6 months *did* exhibit higher levels of proliferation [33]. This, however, has been interpreted rather as a maintenance effect—in other words a prevention of the age-related decrease, in contrast to the acute stimulation of proliferation seen in the current study. 

In C57BL/6 mice, an increase in new-born neurons was observed at 4 and 6 weeks when the bulk of progenitors which were stimulated by running activity during the first week had matured—as expected in this strain [6,7,9]. In DBA/2 animals, we observed an increase in new-born neurons at three weeks, but not at 4 weeks or 6 weeks. Thus it appears that the selection of new-born immature neurons is also impaired in this strain. We hypothesize that the increased number of labeled neurons present at 3 weeks are predominantly at the immature stage [2] and that these, in the absence of the necessary survival signal, die before the fourth week [26]. In C57BL/6, wheel running appears to exert a pro-survival effect in addition to the effect on proliferation as evidenced by the long-term increase in new-born mature neurons, an effect which has been reported before in this strain [4,35]. It is unclear whether the components of the wheel running stimulus that mediate the increase in precursor cell proliferation might also be responsible for the enhanced survival effect. From our previous results in C57BL/6 [33] we had indeed speculated that exercise might trigger a pro-neurogenic program that is initiated in the proliferating precursor cells and is executed in an autonomous fashion. This idea was supported by the fact that neurogenesis from the cohorts of cells whose division had been induced by the running stimulus continued to be increased after cell proliferation had returned to normal. In contrast, our genetic studies had always suggested that proliferation and survival are regulated to a large degree independently.

Our finding above that long-term survival of new-born neurons may be unaffected by running in DBA/2 would, however, support indications that proliferation and survival are mediated by different mechanisms [4,7]. There is considerable evidence that survival of newborn neurons is promoted by network activity [27,28], triggering survival programs such as those mediated by BDNF [29]. The strain difference observed here supporting the dissociation of the two regulatory steps will allow further dissection of the molecular basis of this effect. 

The cumulative labeling experiment with a 6-week survival time does not necessarily contradict the conclusion that long-term neuronal survival may be impaired in DBA/2. It is possible that the new-born neurons seen to increase at this time point are still immature and destined not to be selected for maturation and long-term survival. The 10-day cumulative labeling protocol, especially as it covers here the delayed pro-proliferative response to running, adds several layers of complexity which limit the conclusions that can be drawn from this single result. Further detailed studies are required to dissect the complex dynamics of the effect of physical activity on the post-mitotic survival of new-born neurons. 

**Figure 5:**
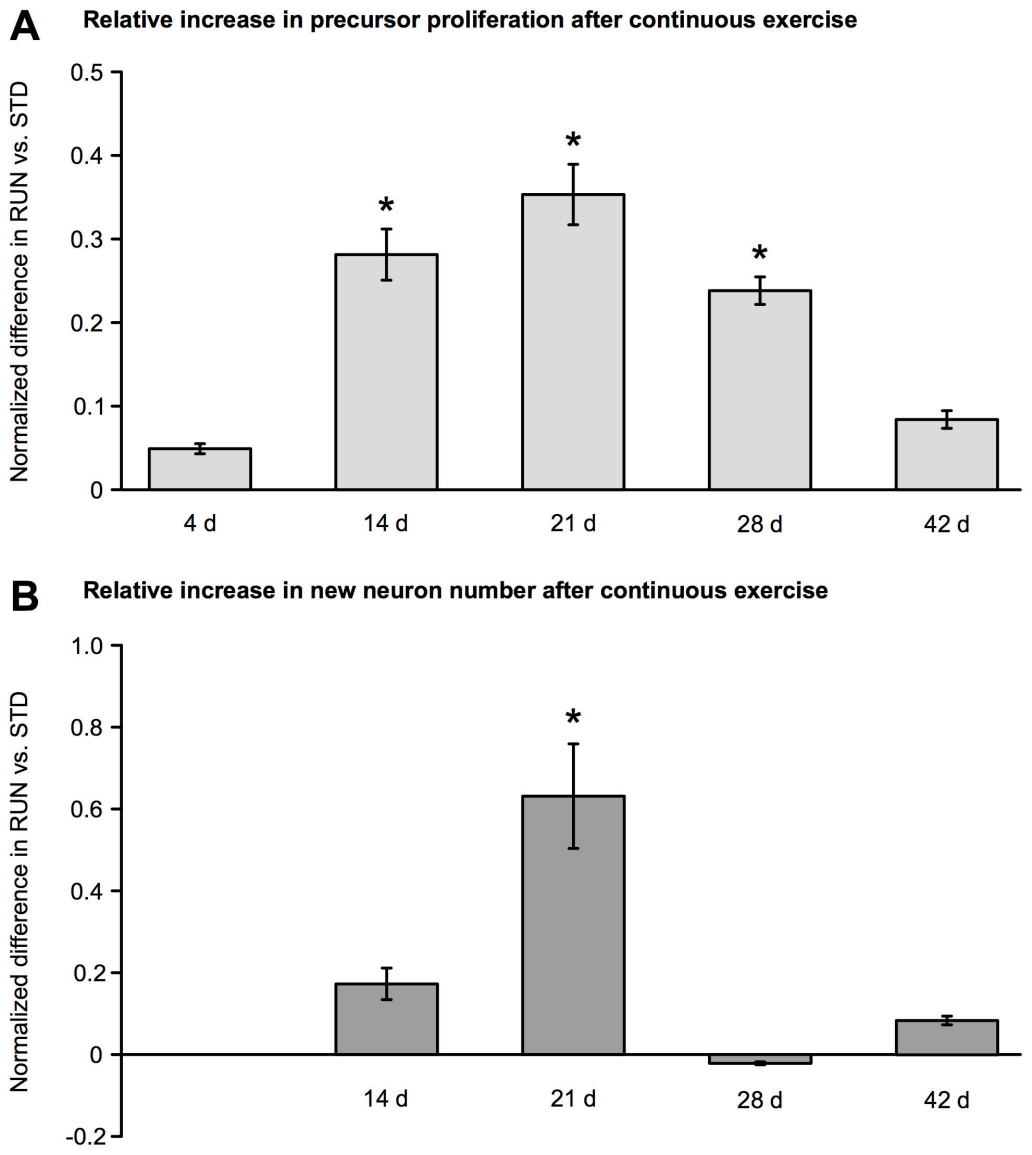
Summary of exercise-induced neurogenesis in DBA/2 over the time course studied. Bars are the normalized difference of means for RUN vs. STD calculated as a ratio of the means. The transient peaks in running-induced proliferation (**A**), and new neuron number (**B**) can be clearly seen. Asterisks indicate a significant (*p* < 0.05) *t*-test from the raw values as presented in the text. Error bars are propagated SEM. See Methods for details.

The experiments described above covered the period in the life of a mouse, in which there is a sharp age-related decrease in baseline precursor cell proliferation [36,37]. This prevents a useful direct comparison of absolute proliferation and new neuron counts between the different time points of our study. However, a helpful overview of the time course of running-induced neurogenesis can be obtained by calculating the normalized difference from control of RUN/STD counts at each time point. The resulting summary graph (Figure **5**) clearly shows the transient peak in running-induced precursor proliferation in DBA/2 mice between around 3–4 weeks and the increase in numbers of new-born neurons observed after 3 weeks of wheel running activity.

Taken together, the data presented above indicate that the effect of wheel running on neural precursor proliferation is different in DBA/2 in comparison to C57BL/6 in a controlled environment with matched groups. Thus, the difference observed between groups could only be due to the genetic background of the animals. As well as being an interesting finding in itself, this phenomenon suggests a possible approach to discovering the genes linking physical activity and precursor proliferation. Adult neurogenesis is a complex phenotype under the control of many genes [3,38]. Without doubt, though, many more genes still remain to be annotated with respect to their role in adult hippocampal neurogenesis. In addition, the position of most known associated genes in the regulatory hierarchy has not yet been established. Genetic resources such as the BXD recombinant inbred panel of mouse strains have suggested potential new components [7] of the regulatory pathway and several such candidates have already begun to be characterized [39,40]. The BXD panel is a powerful tool in the study of adult neurogenesis, because the two progenitor strains, C57BL/6 and DBA/2, exhibit strikingly different baseline rates of proliferation and neurogenesis [6,7]. This phenotypic and genetic variation latent in the progenitor strains is also present, in randomized combinations, within the derived BXD lines. This means that statistically powerful associations between phenotype and genotype can be inferred to help explain the transcriptional control of the process being studied. Thus, the discovery that these two strains also differ in their acute response to physical activity opens up the possibility to draw from the BXD resource to begin uncovering the genetic and molecular mechanisms linking physical activity and increased adult neurogenesis.
